# Design of a Quantitative LC-MS Method for Residual Toxins Adenylate Cyclase Toxin (ACT), Dermonecrotic Toxin (DNT) and Tracheal Cytotoxin (TCT) in *Bordetella pertussis* Vaccines

**DOI:** 10.3390/toxins13110763

**Published:** 2021-10-28

**Authors:** Lisa Szymkowicz, Jeffery Gerard, Benjamin Messham, Wai Wan Vivian Tam, D. Andrew James

**Affiliations:** MS/NMR Center, Analytical Sciences, Sanofi Pasteur Ltd., Toronto, ON M2R 3T4, Canada; lisa.szymkowicz@sanofi.com (L.S.); benjamin.messham@sanofi.com (B.M.)

**Keywords:** *Bordetella pertussis*, adenylate cyclase toxin, dermonecrotic toxin, tracheal cytotoxin, pertussis toxin, quantitative LC-MS, mass spectrometry, vaccine

## Abstract

The antigens for acellular pertussis vaccines are made up of protein components that are purified directly from *Bordetella pertussis (B. pertussis)* bacterial fermentation. As such, there are additional *B. pertussis* toxins that must be monitored as residuals during process optimization. This paper describes a liquid chromatography mass spectrometry (LC-MS) method for simultaneous analysis of residual protein toxins adenylate cyclase toxin (ACT) and dermonecrotic toxin (DNT), as well as a small molecule glycopeptide, tracheal cytotoxin (TCT) in a Pertussis toxin vaccine antigen. A targeted LC-MS technique called multiple reaction monitoring (MRM) is used for quantitation of ACT and TCT, which have established limits in drug product formulations. However, DNT is currently monitored in an animal test, which does not have an established quantitative threshold. New approaches for DNT testing are discussed, including a novel standard based on concatenated quantitation sequences for ACT and DNT. Collectively, the method represents a “3-in-1” analytical simplification for monitoring process-related residuals during development of *B. pertussis* vaccines.

## 1. Introduction

Pertussis disease, commonly known as whooping cough, is a highly infectious respiratory disease caused by the bacterium *Bordetella pertussis*. After initial infection and cold-like symptoms the disease may progress to a phase characterized by a paroxysmal fit of coughing followed by a distinctive “whooping” inhalation breath; in infants and young children the disease can be lethal. The first effective vaccines for prevention of this disease were developed nearly a century ago in the 1930s using inactivated whole-cell *B. pertussis* bacteria [1,2]. In the 1980s, due to the reactogenicity of this type of vaccine and increasing vaccine hesitancy, whole cell pertussis (wP) vaccines were supplanted in Western nations with acellular pertussis (aP) vaccines comprised of purified antigenic protein components from the bacterium [3,4,5]. Despite the long history of both the wP and aP vaccines, the disease remains a major public health concern, with nearly 60 thousand deaths (2015) and the largest disease burden among children less than five years in developing countries [6,7]. Neither infection or vaccination confers life-long immunity [8] and current recommendations include five vaccinations before age seven and a booster shot between ages 11 and 18 [9]. Acellular vaccines are as efficacious as wP vaccines; however, the protection provided by acellular vaccines declines faster [10]. This has increased interest in developing new Pertussis vaccines [11,12,13].

The aP vaccines are typically comprised of protein antigens purified from *B. pertussis* cultures, including pertussis toxin (PT) and other virulence adhesin proteins such as fimbria, pertactin, and filamentous haemagglutinin [14,15]. Because the antigens are produced from fermentations of the causative agent, there are other toxins that can co-purify; it is important that these additional toxins are controlled during development and refinement of purification processes for aP vaccines. These process related impurities include adenylate cyclase toxin (ACT) [16,17] and dermonecrotic toxin (DNT) [18,19], as well as the secondary metabolite tracheal cytotoxin (TCT) [20,21,22]. It is important to monitor these residual toxins and show clearance after antigen purification [23,24].

Both the European Pharmacopeia (Ph. Eur) and World Health Organization (WHO) provide testing and limit guidelines for these residual toxins (Supplement S1). As per Ph Eu. 1356 [23], the ACT concentration must be less than 500 ng per dose, determined by immunoblot analysis or other suitable methods. This can be achieved using an Enzyme-Linked Immunosorbent Assay (ELISA) or using a cell-free enzymatic method [24]. The same EU guideline provide limits for TCT concentration, which must be less than 2 pmol per dose as determined by a biological assay or liquid chromatography (LC). However, unlike ACT and TCT, where regulatory limits have been quantitatively defined, the current regulatory standard for DNT involves an in vivo mouse test for Absence of Residual DNT with a simple pass/fail criterion. Though the pharmaceutical and vaccine industries strive towards reduction, refinement, and replacement of animal safety tests [25,26], to date no in vitro alternative has been accepted by health authorities for DNT testing, and it will continue to pose a challenge until a quantitative limit can be defined.

**Figure 1:**
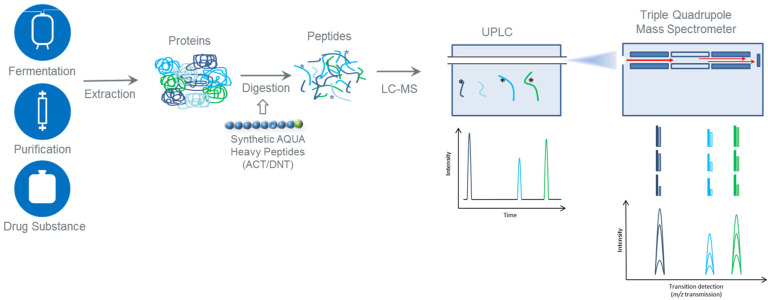
LC-MRM workflow for quantitation of residual toxins from *B. pertussis* across manufacturing stages of aP vaccines. Schematic includes protein extraction, digestion, separation by UPLC and MRM on a Xevo^TM^ Tandem Quadrupole-Stepwave (TQ-S) mass spectrometer. Solutions containing purified TCT standard are run using the MRM method to generate a calibration curve.

Previously, we reported the development of a targeted nano-LC tandem mass spectrometry (MS/MS) method for quantitation of ACT and DNT residual toxins from *B. pertussis* [27]. Herein we describe the design and qualification of a higher throughput LC-MS/MS method for detection and quantitation of ACT, DNT, and TCT. For protein toxins ACT and DNT, a surrogate peptide approach was used with stable isotopically labelled Absolute Quantitation (AQUA^TM^) standards for protein quantitation. However, TCT presented a different challenge because it is a secondary metabolite and not a protein. Although traditional organic synthesis of TCT is possible [28], this approach is not as convenient as isotopically labelled AQUA peptide sequences using standard heavy labelled amino acids. Hence, for TCT, we describe the development and qualification of a more traditional, small molecule quantitation approach using an external calibration curve. Implementation of high flow ultra-performance liquid chromatography (UPLC) coupled to multiple reaction monitoring (MRM) enabled simultaneous monitoring of all three residual toxins pertinent to the safety of aP vaccines using a single analytical workflow (Figure 1).

## 2. Results

### 2.1. TCT Detection in B. pertussis Supernatant Harvest

**Figure 2:**
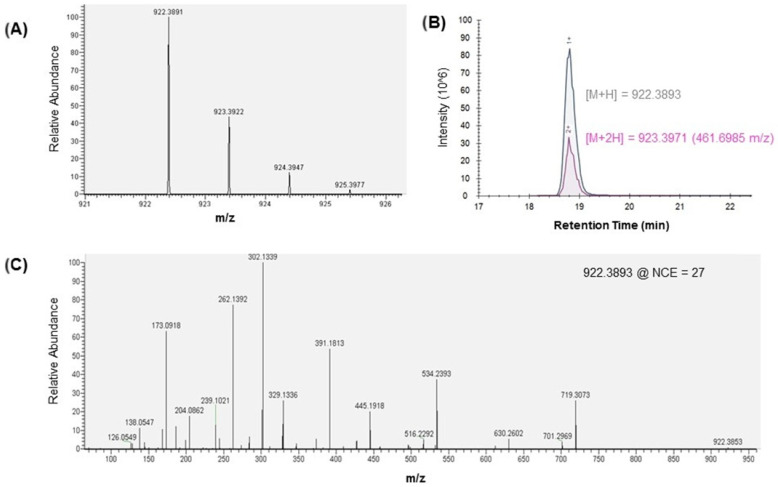
TCT detection in *B. pertussis* supernatant harvest digest. (**A**) High-resolution MS1 survey scan, (**B**) Extracted ion chromatogram (XIC) for singly and doubly charged precursors, and (**C**) MS/MS generated from high-energy C-trap dissociation (HCD) of the singly charged TCT precursor. Refer to Supplement S6 for TCT fragment ion annotation.

Tracheal cytotoxin was detected via nanoLC-MS/MS analysis of *B. pertussis* supernatant harvest digest. The singly charged [M + H] TCT precursor was isotopically resolved in the high-resolution mass spectrometry (HRMS) survey scan (Figure 2a). The monoisotopic peak detected at 922.3891 m/z was a close match to the theoretical [M + H] mass of 922.3893 m/z.

Retention times variability of the TCT peak on reverse phase nano-LC C18 was observed between acquisitions. This could be attributed to weak analyte binding to C18 with elution at approximately 6% (*v/v*) mobile phase B (0.1% FA in ACN). Additional specify was confirmed by co-elution of the singly and doubly charged TCT precursor ions at 922.3893 m/z and 461.6985 m/z, respectively (Figure 2b), as well as MS/MS analysis (Figure 2c). Ionization under nano-electrospray ionization (ESI) conditions favored the singly charged [M + H] precursor with approximately 2.5-fold higher intensity in the extracted ion chromatogram (XIC) of [M + H] versus [M + 2H]. Monitoring the ratio of singly to doubly charged precursor was determined to be critical to developing a robustness quantitation strategy for TCT.

### 2.2. MS/MS Analysis of TCT

**Figure 3:**
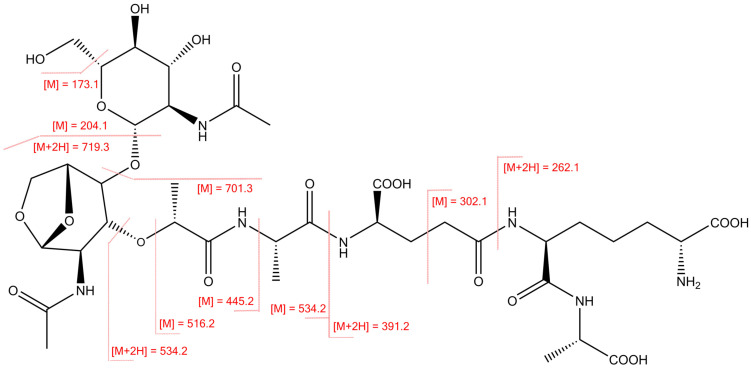
TCT structure and fragment ions generated from high-energy collision induced dissociation (HCD) of the singly charged [M + H] precursor ion. Numerical mass values resulting from cleavage along bonds (in red). The “+2H” notation indicates a proton transfer to the observed ion.

Tandem MS fragmentation by high-energy C-trap dissociation (HCD) generated similar fragment ions to those previously observed by fast atom bombardment mass spectrometry (FAB-MS) of TCT from *B. pertussis* [29,30]. Since HCD fragmentation applies a normalized collision energy based on precursor m/z, a lower collision energy was applied to fragment the doubly charged [M + 2H] precursor at 461.6985 m/z versus the singly charged [M + H] precursor at 922.3893 m/z. This resulted in predominant detection of peptide backbone fragments from the singly charged [M + H] precursor and glycan fragments from the doubly charged [M + 2H] precursor, including the signature O-linked N-acetyl glucosamine (O-GlcNAc) ion series at 204, 186, 168, 144, 138, and 126 m/z [31]. All detected MS/MS fragment ions were singly charged (see fragment ion table in Supplement S6). Fragmentation at oxygen-linked glycosidic and N-terminal to amide bonds yielded transfer of a proton to the observed ion (Figure 3). Dissociation in the peptide portion of the molecule yielded C-terminal fragments at 719.3073 m/z, 701.2969 m/z, and 534.2393 m/z indicating loss of N-acetylglucosamine, GlcNAc + oxygen, and the entire 1-6-anhydro disaccharide with GlcNAc + N-acetylmuramic (MurNac), respectively.

The MS/MS fragmentation pattern observed by HCD (Figure 2c) was similar to that obtained by FAB-MS of TCT from *B. pertussis* [29]. The most abundant fragment ion in the MS/MS spectra from singly charged TCT precursor was 302.1339 m/z (Figure 2c). A fragment ion at 302 m/z was previously detected in FAB tandem mass spectrometry of biologically active peptidoglycan monomers from *Neisseria gonorrhoeae* [32]; however, the 302 m/z fragment ion had not been assigned to the TCT sequence. Analysis of the high-resolution MS/MS spectra generated from HCD fragmentation yielded an assignment of this ion to a fragment of the peptide backbone (Figure 3). Other peptide fragments consistent with previously published unit resolution MS/MS spectra [30,32] include the Ala-DAP ion at 262.1392 m/z and the Ala-DAP-Glu fragment at 391.1813 m/z.

### 2.3. Multiple Reaction Monitoring (MRM) Analysis

A MRM method was designed for targeted quantitation of ACT, DNT, and TCT. Surrogate peptides for ACT and DNT were leveraged from the previously published Parallel Reaction Monitoring (PRM) method [27], with one sequence for quantitation and a second for specificity (Table 1**)**. Given similar collision-induced fragmentation mechanisms on Orbitrap and Triple Quadrupole mass spectrometers, it was expected that the highest responding fragment ions from PRM experiments on the Q Exactive HF Orbitrap would be applicable to MRM experiments on the Xevo^TM^ TQ-S.

One key difference between PRM and MRM methods, is the use of a fixed normalized collision energy in PRM versus the ability to fine tune collision energy (CE) voltage for each fragment ion in MRM. CE voltage is known to impact fragmentation patterns observed for glycopeptides, denoted by significantly different fragmentation patterns for singly and doubly charged TCT precursors using normalized CE in nanoLC-MS/MS.

Optimization of CE was performed in three iterative rounds for the top three transitions per ACT/DNT/TCT precursor identified by PRM. Synthetic ACT/DNT AQUA peptides standard (20 fmol/µL) and purified TCT standard (20 ng/mL) were used to refine CE values in the MRM method. First, crude optimization of CE values was performed for TCT with 10 V steps applied to the singly and doubly charged precursors from 13 to 53 V for [M + H] and 6 to 46 V for [M + 2H] precursor masses. Second, stepped CE was performed in 1 V increments ramping 4 V above and below the Skyline-predicted CE values for ACT and DNT, and ± 4 V from the values obtained for TCT in round 1. Lastly, CE values were optimized by ramping ± 4 V from CE value identified as giving highest peak area response in round 2. Following CE-optimization, new heavy and native MRM methods were created with the top two transitions per peptide listed in Table 1.

The LC was reconfigured for MRM; switching from nano LC run at 300 nL/min with a 75 μm column internal diameter for PRM, to high-flow UPLC run at 300 μL/min with a 2.1 mm internal diameter. The use of high-flow chromatography provided several benefits, including much shorter acquisition methods, more robust system performance, and better chromatographic behavior for TCT, while maintaining the required sensitivity for detection of the ACT and TCT below the Ph. Eu limits [33].

### 2.4. Quantitation by Multiple Reaction Monitoring (MRM)

The MRM workflow (Figure 1) combined MS-based protein quantitation using surrogate AQUA peptide standards for ACT and DNT, with traditional small molecule quantitation using an external standard curve for TCT. Performance metrics were re-assessed in the MRM method using multiple isotopologue reaction monitoring (MIRM) [34]. The mass resolution setting for the 1st quadrupole (Q1) was adjusted from unit mass resolution with full width at half maximum set to 0.75 (FWHM = 0.75) to a custom MIRM setting (FHWM = 0.5). On tandem quadrupole MS instruments, peptide signal response is inversely proportional to the quadrupole resolution. Therefore, increasing the quadruple resolution decreases the number of ions passing through the instrument (i.e., signal response) but increases specificity for a given precursor-fragment ion transition. Higher specificity is particularly advantageous for complex sample matrices and doubly charged ACT/DNT/TCT peptide precursors with naturally occurring isotopes spaced 0.5 m/z apart.

The ACT/DNT AQUA peptides and TCT standard were spiked into a purified PT antigen, and theoretical isotopic abundances calculated for the most abundant transition per peptide. AQUA peptide signal response was determined to be linear with an R^2^ ≥ 0.99 from 1 to 100 fmol on-column (Supplement S7). The limits of quantitation (LOQ) were determined based on the lowest abundance MIRM channel with S/N ≥ 10 and measured abundance within 20% of the theoretical isotopic abundance. The limits of detection (LOD) were estimated based on the lowest abundance MIRM channel with S/N ≥ 3 across triplicate measurements (Table 2).

Dose-response linearity for TCT was evaluated from plots of peak area vs. TCT concentration (ng/mL) for an external calibration curve. Comparison of linear versus quadratic fit showed no significant difference in correlation coefficient (R^2^) or residuals over three orders of magnitude from 5 to 531 pg TCT (Supplement S8). Using a linear fit, a bias was observed when the calibration range was extended to 4 orders of magnitude. This result was consistent with previous published work suggesting that quadratic fit and 1/X^2^ weighing is most suitable for bioanalytical LC-MS/MS assays [35]. Use of a quadratic fit resulted in an R^2^ value of 0.9929 from 5.31 to 5314 pg TCT (Supplement S8).

Results of TCT signal response from the external calibration curve revealed that the doubly charged TCT precursor was 3 to 4.5-fold higher in response than the singly charged precursor under high-flow ESI conditions. This result was contrary to previously described PRM results, which demonstrated preferential ionization of the singly charged TCT precursor under nano-ESI conditions. The proportion of singly changed TCT precursor was observed to increase at higher concentrations of TCT. Processing the sum of TCT response from singly and doubly charged precursors lowered the measured variability (i.e., lower % coefficient of variation (%CV)) between replicate injections (supplement S9). To facilitate robust quantitation of TCT, transitions with a common fragment from both the singly and doubly charged TCT precursors were summed in the final MRM method. The fragment ion resulting from loss of GlcNAc was selected as it produced the highest response in both charge states following CE optimization on the Xevo^TM^ TQ-S (i.e., 922.3893 > 719.3099 + 461.6985 > 719.3099 m/z). No endogenous ACT, DNT, or TCT were detected at the purified PT antigen drug substance (DS) stage using the finalized LC-MS/MS (MRM) method.

### 2.5. Method Qualification

Method performance for ACT and TCT quantitation was evaluated by spiking recombinant ACT standard and purified TCT into purified PT antigen at concentration levels spanning the Ph. Eu. 1356 regulatory limits (Table 3). Method accuracy was assessed by determination of percent recovery from the measured ACT/TCT concentration divided by the theoretical amounts spiked into each sample. The recovery ranged from 81% to 89% for ACT and 114% to 128% for TCT (Table 3).

Both repeatability and intermediate precision were assessed at three spike concentration levels for ACT in PT antigen DS. For repeatability at each level, a total of nine determinations obtained across three experimental days were used to calculate a pooled standard deviation. The pooled standard deviation was used to calculate a standard error based on two independent preparations in the reportable value. The %CV for repeatability of ACT quantitation using NIENAVGSAR was 2.3% to 4.1% for 50.0 to 600.0 ng ACT per dose. The %CV values for intermediate precision were ≤ 7.8% for ACT using NIENAVGSAR for quantitation over the range described above. Precision of TCT quantitation was evaluated for five spike concentration levels spanning 20% to 638% of the EU limit of 2 pmol per dose (Table 3). The %CV values for repeatability were 0.9% to 5.7% for 0.4 to 12.8 pmol of TCT per antigen dose. The %CV values for intermediate precision were 4.0% to 12.0% for 0.4 to 12.8 pmol of TCT per dose.

**Figure 4:**
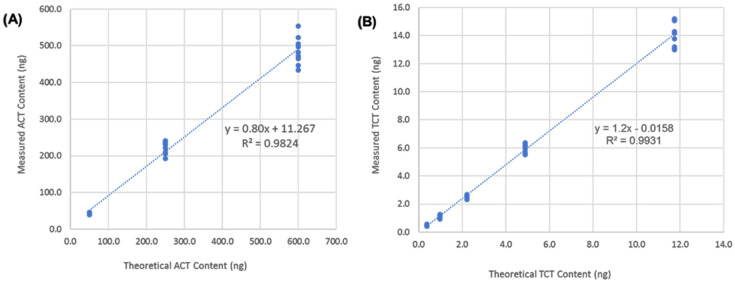
Method linearity for (**A**) ACT and (**B**) TCT spiked into PT antigen drug substance samples. Results plotted for n=9 determinations per concentration level.

Method linearity was evaluated by plotting measured versus theoretical toxin concentration across the spike levels. Results for ACT content were plotted for nine determinations at three concentration levels, yielding a correlation coefficient (R^2^) of 0.9824 for 50 to 600 ng ACT using NIENAVGSAR for quantitation. Linearity was also confirmed for TCT with an R^2^ of 0.9931 for 0.3684 to 11.7 ng TCT, equivalent to 0.4 to 12.8 pmol per dose. The slope values for ACT and TCT were within the range of 0.8–1.2 (Figure 4), in line with the percent recovery results for method accuracy (Table 3). The lower limits for quantitation (LLOQ) were calculated based on 10 times the pooled standard deviation from *n* = 9 determinations of the lowest spike level. The method LLOQ values of 14.1 ng and 0.41 pmol were well below the EU regulatory limits of 500 ng and 2 pmol per dose, respectively, for ACT and TCT.

## 3. Discussion

This work was initiated to develop a quantitative method for routine analysis of ACT and TCT in aP vaccine antigens at the DS stage, as these toxins have established analytical safety thresholds. The DNT peptide sequences were maintained as standards in the method to allow for in-process monitoring of all three *B. pertussis* residual toxin within a single workflow.

Prior to development of an LC-MS workflow, analysis of residual *B. pertussis* toxins required three separate methods. This includes an ELISA or enzymatic test for ACT [24], an HPLC assay for TCT, and animal test for DNT [33]. Development and maintenance of three separate assays is complex and inefficient. In addition, matrix changes may have different effects on the individual assays making analysis of process intermediates challenging to demonstrate toxin clearance. A single quantitative LC-MS method that can detect and quantitate all residual toxins in one workflow would improve efficiency and streamline analysis, testing, and reporting. A total content method is also inherently more stringent than an activity-based assay as it will detect inactive forms of the toxin(s).

We designed this new method by adapting the targeted analysis work reported by Szymkowicz et. al. [27] describing a PRM workflow for protein toxins, ACT and DNT, on a Q-Exactive HF nanoLC-MS/MS system. For the development of a routine test, several additional requirements are typically considered, including system sensitivity, system reliability, assay throughput, ease of analysis, and LC-MS system compliance requirements, which are governed by good manufacturing practices. Given these considerations, we decided to build our quantitative method on a Waters Xevo^TM^ TQ-S coupled to an H-Class^TM^ UPLC; a tandem quadrupole system, using standard analytical flow rates and columns. By moving to an analytical flowrate system, we were able to reduce our method acquisition time from 95 min to 14.5 min per experiment while maintaining similar overall assay sensitivity.

Protein quantitation by MRM is an established workflow used in academic, clinical, and industrial settings [36,37]. MRM is a targeted workflow that allows for selection of a precursor and fragment pair, called a transition, that is specific to the MS detection of the analyte of interest based on its molecular structure. MRM workflows can be designed for any biomolecule, including peptides and small molecules. The specificity of detection for a given analyte can be increased by selecting multiple transitions for each molecule. For our residual toxin quantitation method, we selected two transitions per analyte; one transition to quantitate, the second transition to confirm specificity.

To quantitate proteins by MRM, samples must first be digested using a protease with specific and defined cleavage characteristics. The proteolytic peptides produced during sample digestion are detected in the LC-MS system, providing greater specificity, sensitivity, and accuracy of analysis to the method. It is these peptides, released during sample digestion, that are measured and quantitated during analysis; protein amounts are then inferred.

Tracheal cytotoxin (TCT) is a small glycopeptide released from the *B. pertussis* cell wall during the logarithmic phase of cell growth [20]. The biomolecule is 921 Da and can be detected directly by MS, however, upstream sample processing (e.g., protein precipitation) is typically used to remove complex protein matrices prior to reverse-phase HPLC (RP-HPLC) separation. Recently, a hydrophilic interaction liquid chromatography (HILIC)-MS method was described for quantitation of TCT in pertussis vaccines [38]. Use of HILIC separation yielded improved chromatographic performance, however, the strong organic solvents would not be amendable to analysis of protein toxins, ACT and DNT. In our method with RP-HPLC coupled to MRM, we detect TCT after enzymatic digestion to allow for measurement of all three residual toxins in one workflow (refer to MRM method described in Section 5).

The MRM method was qualified from 50 to 600 ng for ACT and from 0.4 to 12.8 pmol for TCT, well below the EU regulatory limits of 500 ng per dose for ACT, and 2 pmol per dose for TCT [33]. It should be noted that the regulatory limits for ACT and TCT are expressed per dose at the drug product (DP) stage, whereas testing for residual toxins is performed at the purified DS stage prior to the final formulation with adjuvant. Typical doses for aP vaccines can range from 3 to 20 µg per antigen component. To address the DS-to-DP conversion, the amount of toxin is reported per µg of antigen. A simple calculation is then performed based on the target dose of the specific DP formulation. This approach will allow for broader method applications, where any subset of aP antigens can be tested at the purified DS stage and then the total residual toxin values can be summed according to a specific product formulation(s).

For TCT, we used an external standard that was purified from *B. pertussis* culture supernatant [29]. In the results, we also included a tandem mass spectrum of TCT (Figure 2) derived from nano LC-MS/MS analysis on the Q-Exactive HF to provide updated fragment assignments supporting transition selection for this analyte in our MRM method. This high mass accuracy MS/MS spectra also allowed us to identify novel fragment features for TCT providing new information for this peptidoglycan component released from the *B. pertussis* cell wall (Figure 3).

In our sample preparation workflow (Figure 1) we added AQUA heavy peptide standard for ACT and DNT to samples prior to proteolysis with Trypsin/Lys-C. Spike-recovery experiments with ACT and TCT reference standards showed the accuracy and precision of the workflow for these two toxins (Table 3). We also demonstrated that TCT was recovered through the sample preparation protocol (Section 5.3), with no impact from the digestion required for analysis of protein toxins ACT and DNT.

Protein quantitation using AQUA peptide standards depends on the accuracy of the heavy-labelled synthetic peptides provided by the vendor, as well as the signal stability of both native and synthetic peptides sequences [39]. As the quality requirements increase for an assay, so do the requirements for monitoring the critical reagents in the assay. In-house approaches will be required to monitor the stability of AQUA peptide reagents over time. This may present a number of challenges, as AQUA peptides are typically provided at 5 pmol/µL, which is near LOQ for most LC-based amino acid analysis (AAA) workflows. Vendors typically circumvent quantitative limits for AAA by testing purified bulk synthetic peptides, then diluting and aliquoting before shipment to the customer. Quantitative analysis of final stocks is not typically performed. In addition, AAA is not an ideal method to monitor peptide stability as the intact molecule is first hydrolyzed in acid to individual amino acid constituents. New approaches will need to be considered as AQUA peptide reagents are implemented in QC batch release tests. RP-HPLC with UV detection could be used to monitor AQUA peptide reagents [40]. Quantitative Nuclear Magnetic Resonance (NMR) may also be an interesting option [41], although access to a system with the desired capabilities could limit widespread use.

Analysis of DNT remains challenging since there is currently no quantitative threshold established for DNT. Existing regulations refer only to the absence of neurotic lesions when mice are administered the equivalent of one vaccine dose [33]. A broader discussion between vaccine manufacturers, regulators, and academic researchers will be needed to establish a quantitative threshold for DNT to support development of alternative in vitro methods [26].^.^ No certified analytical reference standard is currently available for DNT; however, a recently published protocol described production of recombinant DNT that could facilitate screening of alternative cell or immunochemical-based approaches, in addition to quantitative accuracy/precision in MS-based workflows [42]. The detection limit for LC-MS analysis of DNT (Table 2) would need to be improved by an estimated 10 to 100-fold before MRM workflows can approach the sensitivity of current animal methods.

One strategy that may be able to address the lack of a DNT reference standard is the use of a Quantification conCATamer (QconCAT) construct for MS-based quantitation. QconCAT is an artificial recombinant protein made up of linked, digest-specific, peptide sequences from several proteins [43]. Flanking residues from the native protein sequences can be inserted around the concatenated peptides, to replicate digestion kinetics experienced by protein(s) of interest [44]. These constructs can be expressed in *E. coli* and spiked into evaluation samples, prior to digestion, to assess method performance metrics such as accuracy and precision across a relevant concentration range. Alternatively, the constructs can be expressed in N^15^ enriched media to add a metabolic heavy label into every amide bond in the QconCAT sequences. N^15^ heavy labelling would allow the QconCAT to be used as an internal quantitation standard [45] in lieu of AQUA peptide standards for ACT and DNT.

## 4. Conclusions

In this work, we developed an in vitro method for detection and quantitation of residual *B. pertussis* toxins, ACT, DNT, and TCT, using a targeted LC-MS (MRM) method. The residual toxin method described by our group previously [27] was transferred to a tandem quadrupole system to facilitate routine analysis requiring higher throughput and the addition of an external calibration curve for TCT. A fully quantitative method is described for ACT and TCT, while DNT detection is currently suitable as a limit test. Method sensitivity was determined to be adequate for monitoring clearance of ACT and TCT below established pharmacopeia limits [23,33]. Additional work is required to establish a quantitative threshold for DNT and increase sensitivity for DNT peptide detection. Collectively, the work highlights qualification of an LC-MS test for residual toxins in a PT antigen, with potential for broad application in the testing and characterization of all aP vaccines. 

## 5. Materials and Methods

### 5.1. Standard Preparation

Recombinant ACT for spike-recovery studies was purchased from List Biologics (Campbell, CA, USA Cat #198L). AQUA peptide standards were custom ordered from Thermo Fisher (Rockford, IL, USA) based on the surrogate peptide sequences selected for nano LC-MS/MS analysis of ACT and DNT in Szymkowicz et. al. [27]. One tryptic peptide was selected for absolute quantitation and a second sequence for confirmation of identity. Each AQUA peptide was synthesized with a ^15^N, ^13^C labelled terminal arginine and spiked into protein samples internal standards prior to digestion. Amino acid analysis (AAA) was performed by the vendor on the stock solutions. Peptides were provided in individual aliquots diluted to 5 pmol/µL in 5% acetonitrile (ACN) in water.

The TCT reference material was obtained from the Goldman Laboratory (University of North Carolina), following purification from culture supernatant of *B. pertussis* strain Tohama I or III as described by Cookson et. al. [29]. The TCT standard material was characterized by RP-HPLC and shown to be free of peptide contaminants. Starting concentrations were determined using amino acid analysis.

### 5.2. Nano LC-MS/MS Analysis of TCT in B. pertussis Supernatant Harvest

Supernatant harvest from *B. pertussis* Tohama I strain was digested with Trypsin/Lys-C as previously described [27]. Prior to nano LC-MS/MS analysis, digest samples were thawed at room temperature and then transferred to low-volume autosampler vials (Waters p/n 186005663CV). Nano LC-MS/MS experiments were run on a Q Exactive High Field (HF) mass spectrometer with an UltiMate 3000 RSLCnano UHPLC system (Thermo Scientific, Bremen, Germany).

The targeted parallel reaction monitoring (PRM) acquisition method previously reported for ACT and DNT [27] was used as a starting point for TCT analysis. 2 µg of *B. pertussis* supernatant harvest digest was loaded onto a C18 trap column (µ-Precolumn 5 mm × 300 µm internal diameter (ID), Thermo Cat #16054) at 5 µL/min with 99% A/1% B (0.1% trifluoroacetic acid (TFA) in water/ACN). After 2 min of on-line desalting/pre-concentration, the flow was switched to an EASY-Spray column, 15 cm × 75 µm ID, PepMap C18, 3 µm analytical column with integrated ESI emitter (Thermo Cat #ES800). Peptides were eluted at 300 nL/min with a 95-min gradient from 1 to 35% 0.1% formic acid (FA) in ACN. The total run time was 117 min with the column temperature set to 45 °C for the Trap column and 30 °C for the analytical column. The targeted PRM acquisition method cycled between one Full MS survey scan and 15 MS/MS scans with a 1.4 m/z isolation window. Full MS survey scans were acquired from 380 to 2000 m/z with a resolution of 120,000, automatic gain control (AGC) of 3 × 10^6^, and maximum injection time (maxIT) of 50 ms. MS/MS fragmentation was performed during the PRM scans using a resolution of 120,000, normalized collision energy (NCE) of 27, maxIT of 300 ms, and AGC of 2 × 10^5^. ESI source conditions include capillary voltage of 1.9 kV, capillary temperature of 275 °C, and S lens RF voltage set to 50.0.

### 5.3. Sample Preparation for LC-MRM

The ACT and DNT AQUA peptides listed in Table 1 were pooled to a concentration of 1 pmol/µL (per peptide sequence) stabilized in a background of 2 pmol/µL enolase digest (Waters p/n 186002325). To normalize for batch-to-batch variation in AQUA peptide concentration, a concatenated (QconCAT) protein construct [43,45] was designed containing ACT and DNT surrogate peptides with six flanking residues from the endogenous protein sequences and a C-terminal histidine tag (refer to Supplement S2). A control sample was prepared with 10 μg/mL of native QconCAT in MassPrep Protein Standard Mix (Waters 186004900). The QconCAT control was digested alongside the samples and analyzed by LC-MS/MS (MRM). The reference AQUA peptide concentrations were calibrated based on the concentration of QconCAT measured by LC-MS/MS (MRM) versus theoretical concentration determined by bicinchoninic acid (BCA) assay [46].

A mastermix was prepared with the following reagents: 15 μL of 1 M ABC (pH 7.4 to 7.5), 15 μL of 1% RapiGest^TM^ (*v/v*, Waters, p/n #186001860), 1.5 μL of 500 mM tris(2-carboxyethyl) phosphine solution (Thermo Scientific, Cat #77720), 15.0 μL of 200 mM chloroacetamide (Thermo Scientific A39270) and 3.12 μL of pooled ACT/DNT AQUA peptide stock solution. 25 μg of purified antigen was added to the mastermix and completed to 145 µL with LC-MS grade water. Each sample was digested in duplicate. Samples were reduced/alkylated by incubating at 95 °C for 10 min in a thermomixer set to 300 rpm. The reduced and alkylated samples were enzymatically digested by adding 5.0 μL of 0.5 μg/μL Trypsin/Lys-C proteolytic mixture [47] for 3 h at 37 °C using a thermomixer incubator set to 300 rpm for mixing. The digests were quenched with the addition of 6.0 μL of TFA (Pierce, Cat #28904), bringing the total digest volume to 156 μL and theoretical concentration of AQUA peptides standards to 20 fmol/μL per peptide. The acidified digest samples were confirmed to have a pH of ∼2 using a pH strip and were then incubated at 37 °C for 30 min then centrifuged at 8100× g for 10 min to facilitate RapiGest^TM^ hydrolysis [48]. Digest supernatants were transferred to new tubes and stored at −80 °C until LC-MS analysis.

### 5.4. LC-MRM Experimental Design

The availability of high-confidence MS/MS spectra from previous work on the Q-Exactive HF Orbitrap Mass Spectrometer (Thermo Scientific, Bremen, Germany) [27] meant that a relatively small number of transitions for each sequence could be selected for implementation of a higher throughput MRM method. The top three fragments identified for each peptide/precursor in previous targeted MS/MS (PRM) experiments were selected for optimization on the Xevo^TM^ TQ-S Mass Spectrometer (Milford, MA, USA).

For spike-recovery experiments, recombinant ACT and purified TCT standard were spiked into PT antigen material at concentrations corresponding to 10%, 50%, and 120% of the EU regulatory limits [33]. Each spike sample was aliquoted, stored at ≤−60 °C, then digested in triplicate on three separate days for a total of nine independent replicates per level.

### 5.5. LC-MRM Data Acquisition

Before MS analysis, sample digests were thawed at room temperature and then transferred to low volume autosampler vials. LC-MS/MS (MRM) experiments were run on a Xevo^TM^ TQ-S MS system with an ACQUITY H-Class analytical UPLC (Waters Corporation, Milford, MA, USA). Experiments were run on a ACQUITY UPLC BEH C18 Column, 130Å, 1.7 µm, 2.1 mm × 50 mm column (Waters p/n 1860002350) with ACQUITY UPLC BEH C18 VanGuard Pre-column, 130Å, 1.7 µm, 2.1 mm × 5 mm (Waters p/n 186003975). As with the nanoLC experiments, mobile phases consisted of water (mobile phase A) and ACN (mobile phase B) with 0.1% FA added as an ion-pairing agent. Peptides were eluted at 0.3 mL/min using 5-min gradient from 1 to 30% 0.1% FA in ACN. The total run time was 14.5 min. Additional MS tune and LC method gradient details are provided in Supplement S3 and S5, respectively.

An external TCT calibration curve was prepared by diluting purified TCT standard in 0.1% FA in water to yield five concentration levels at 1, 10, 40, 70, and 100 ng/mL, respectively. Each TCT calibration level was injected at the beginning and end of the sample set, with the response averaged to generate a calibration curve. All digest samples, standards, and blank injections were analyzed with 1 µL injections. A cleaning run was performed between each set of duplicate samples, consisting of a 10 µL injection of water/acetonitrile/methanol/isopropyl alcohol (1:1:1:1) and three sawtooth gradients oscillating between 1% and 95% B (Supplement S4).

### 5.6. Data Analysis

Raw files from the Themo Q-Exactive HF were analyzed in Qual Browser (Thermo Scientific). Skyline v4.1 was used to refine the CE applied to each transition [49]. After MRM method optimization, data processing was carried out in TargetLynx software (version 4.1, Waters Corporation). For ACT and DNT, the ratio of native-to-heavy peak areas was calculated for each peptide based on the transitions listed in Table 1. Concentrations were determined from the native-to-heavy ratio given the known concentration of heavy peptide standards spiked into the samples. One peptide sequence per protein was used for absolute quantitation and a second for confirmation of identity. The precursor-fragment transition yielding the highest intensity was selected for quantitation in the finalized method (Table 1). For TCT, transitions from the singly and doubly charged precursors were summed to account for variation during ionization. TCT quantitation was performed by comparing signal response in the samples against an external calibration curve.

## Figures and Tables

**Table 1:** Fragment ion transitions for LC-MS/MS (MRM) analysis of ACT, DNT and TCT peptides. Underlined letters indicate incorporation of N^15^ heavy labelled amino acids.

Toxin	Theoretical Intact Molecular Weight (g/mol)	Peptide	Fragment Type	Description	Precursor (m/z)	Fragment (m/z)	Cone (V)	Collision Energy (V)
ACT	177 414	NIENAVGSAR ^A^	y8 *	Native	515.7674	803.4006	35	19
				Heavy AQUA	520.7716	813.4089	35	19
			y7	Native	515.7674	674.3580	35	22
				Heavy AQUA	520.7716	684.3663	35	22
		ITGDAQANVLR ^B^	y9	Native	579.3173	943.4956	35	22
				Heavy AQUA	584.3214	953.5038	35	22
			y5	Native	579.3173	572.3515	35	22
				Heavy AQUA	584.3214	582.3597	35	22
DNT	160 644	ELPALIGASGLR ^A^	y6 *	Native	598.8535	560.3151	35	21
				Heavy AQUA	603.8576	570.3234	35	21
			y7	Native	598.8535	673.3991	35	24
				Heavy AQUA	603.8576	683.4074	35	24
		NDDLVSIAATYDR ^B^	y8	Native	726.8519	896.4472	35	24
				Heavy AQUA	731.8560	906.4555	35	24
			b3	Native	726.8519	345.1041	35	29
				Heavy AQUA	731.8560	345.1041	35	29
TCT	921	C_37_H_59_O_20_N_7_	Peptidoglycan	[M + H]	922.3888	719.3099	35	31
				[M + 2H]	461.6985	719.3099	35	11

^A^ Sequence selected for protein quantitation. ^B^ Sequence selected for confirmation of protein identity (i.e., specificity). * Fragment ion transitions for protein quantitation.

**Table 2:** MS performance metrics for ACT, DNT, and TCT from MIRM evaluation.

Toxin	Standard Type	Peptide Standard	LLOQ ^1^		LOD ^2^	
			On-Column	per 25 µg Digest	On-Column	per 25 µg Digest
ACT	Internal—AQUA peptide standards (20 fmol/µL)	NIENAVGSAR	93.6 amol	2.6 ng (14.6 fmol)	21.4 amol	0.59 ng (3.39 fmol)
		ITGDAQANVLR	116 amol	3.2 ng (18.1 fmol)	63.6 amol	1.76 ng (9.92 fmol)
DNT	Internal—AQUA peptide standards (20 fmol/µL)	NDDLVSIAATYDR	1.96 fmol	49.1 ng (306 fmol)	375 amol	9.4 ng (58.5 fmol)
		ELPALIGASGLR	3.97 fmol	99.5 ng (619 fmol)	655 amol	16.4 ng (102 fmol)
TCT	External—purified TCT standard	Purified TCT	2.3 pg	0.388 pmol (0.36 ng)	0.423 pg	0.069 pmol (63.6 pg)

^1^ MIRM channel with S/N ≥ 10 and measured abundance ± 20% theoretic isotopic abundance value. ^2^ Lowest channel with S/N ≥ 3 across triplicate MIRM measurements.

**Table 3:** Method qualification results for ACT and TCT in spiked PT antigen drug substance.

Toxin	Ph. Eu. 1356 Limit	% EU Limit	Accuracy (% Recovery)	Precision (% CV)		Linearity	Qualified Range (per Dose)	LLOQ (per Antigen Dose)
				Repeatability	Intermediate Precision			
ACT	500 ng per dose	10.0%	87	2.3	5.3	R^2^ = 0.9824	50.0 to 600.0 ng	14.1 ng
		50.0%	89	2.6	7.7			
		120.0%	81	4.1	7.8			
TCT	2 pmol per dose	20.0%	128	5.7	12.0	R^2^ = 0.9931	0.4 to 12.8 pmol	0.41 pmol
		53.1%	118	5.0	9.5			
		120.0%	114	2.6	4.0			
		265.7%	122	1.1	5.2			
		637.7%	120	0.9	6.4			

## Supplementary Materials

The following are available online at https://www.mdpi.com/article/10.3390/toxins13110763/s1, Supplement S1. Ph. Eur. 1356 Limits for Residual Toxins ACT, DNT, and TCT per dose of acellular Pertussis vaccine. Supplement S2. Concatenated QconCAT protein construct containing ACT and DNT peptide sequences. Supplement S3. Tune and inlet settings for the LC-MRM method on a Water Acquity H-class analytical UPLC and Xevo^TM^ TQ-S mass spectrometer. Supplement S4. Triple sawtooth LC gradient for column cleaning between samples. Supplement S5. Optimized MS method for multiple reaction monitoring (MRM) analysis of ACT, DNT and TCT analysis on the Xevo^TM^ TQ-S. Supplement S6. TCT fragment ions from high-energy collision induced dissociation (HCD) on a Q-Exactive HF Orbitrap mass spectrometer. Supplement S7. Dose linearity for ACT AQUA peptide standards. Supplement S8. External TCT calibration curves using the singly and doubly charged fragment ion transitions. Supplement S9. TCT signal response from a multi-sample external calibration curve analyzed by MRM. References [32,45] are cited in the supplementary materials.
