# Supplementary material for: Design of a Quantitative LC-MS Method for Residual Toxins Adenylate Cyclase Toxin (ACT), Dermonecrotic Toxin (DNT) and Tracheal Cytotoxin (TCT) in Bordetella pertussis Vaccines
Source: Toxins (Basel). 2021 Oct 28;13(11):763. doi: 10.3390/toxins13110763 (PMC8624556; doi:10.3390/toxins13110763)
Supplement: Supplementary file 1 [file toxins-13-00763-s001.zip › toxins-1425125-supplementary.pdf]

Supplementary Materials: Design of a Quantitative LC-MS Method for Residual Toxins Adenylate Cyclase Toxin (ACT), Dermonecrotic Toxin (DNT) and Tracheal Cytotoxin (TCT) in *Bordetella pertussis* Vaccines

Lisa Szymkowicz, Jeffery Gerard, Benjamin Messham, Wai Wan Vivian Tam and D. Andrew James

Supplement S1. Ph. Eur. 1356 Limits for Residual Toxins ACT, DNT and TCT per dose of acellular Pertussis vaccine.

Toxin	Abbreviation	Molecule Type	Theoretical Molecular Weight	EU Limit per Dose	Limit in Moles per Dose
Adenylate Cyclase Toxin	ACT	Protein	177 414 g/mol	500 ng	2.8 pmol
Dermonecrotic Toxin	DNT	Protein	160 644 g/mol	Absence	Not Defined
Tracheal Cytotoxin	TCT	Glycopeptide	921 g/mol	1.84 ng	2.0 pmol

Supplement S2. Concatenated (QconCAT) protein construct containing ACT and DNT peptide sequences flanked by six flanking residues from the endogenous protein sequences and a C-terminal poly-histidine tag. Surrogate peptides with matching AQUA peptides denoted with underline. The QconCAT construct was expressed as a native recombinant protein in *E. coli* and purified using a C-terminal His-tag as per the described protocol in Scott *et al.* [1].

```
MGSSGTTSNVLRNIENAVGSARDDVLIGTELADRITGDAQA
NVLRGAGGADAHWGQRALQGAQAVAAAQRLVHAIAGRDTIR
INAGADQLWFARQGNDLEREWVRKASALGVDYYDNVRNVEN     ACT
VIDEMPRKLDAWEIAKFHLAATWIVPFYREIFFSTQDRSYR     DNT
VDVWRSVLKELPALIGASGLRLSRSLLEYVKARYEIYYLLN
RVPHPLAIPAPMRNDDLVSIAATYDRAVIAYLKVRHYKVLT
SQPGLIARGIENHNRLQEVRAYIGDLSPVNDVLYRAGYDLD
IAYLGKDVPGGGSTRITRHDEPVPIRRLVAGFVSATTVGGR
NQAFLR…HHHHHH
```

Supplement S3. Tune and inlet settings for the LC-MRM method on a Water Acquity H-class analytical UPLC and Xevo™ TQ-S mass spectrometer. Mobile phases consist of 0.1% FA in Water (i.e. line C) and 0.1% FA in ACN (i.e. line D).

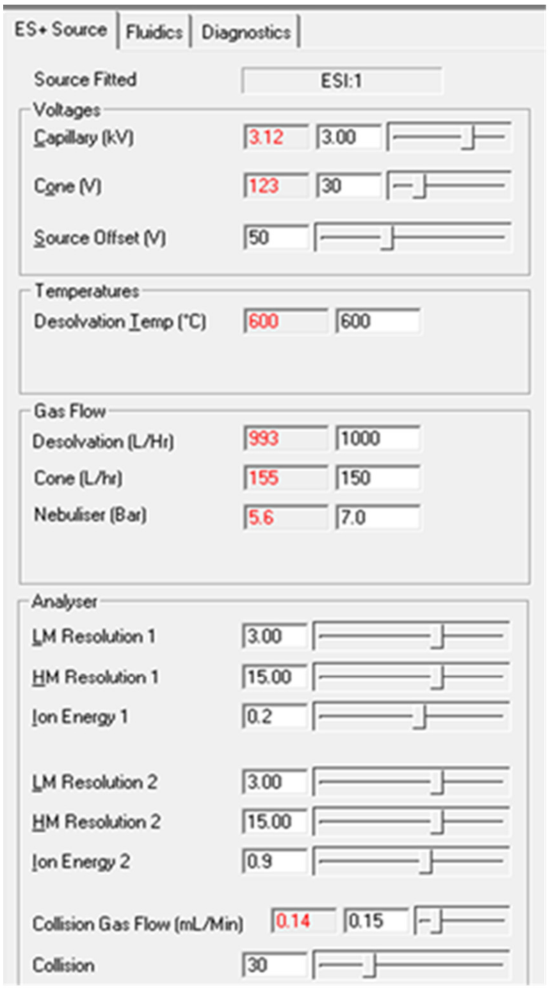

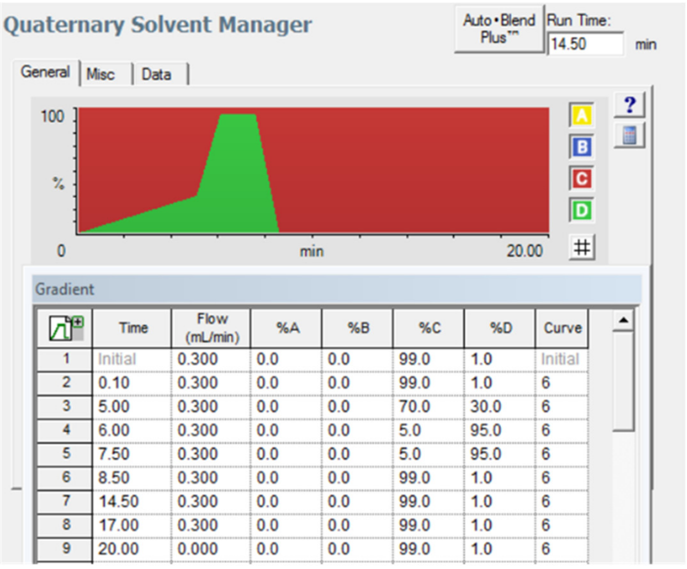

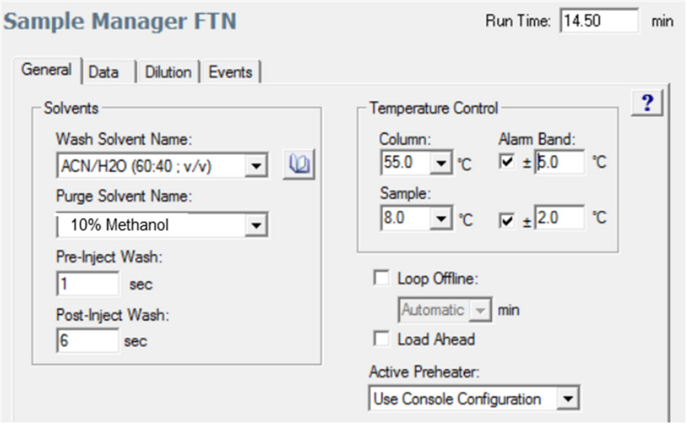

Supplement S4. Triple sawtooth LC gradient for column cleaning between samples. Mobile phases consist of 0.1% FA in Water (i.e., line C) and 0.1% FA in ACN (i.e., line D).

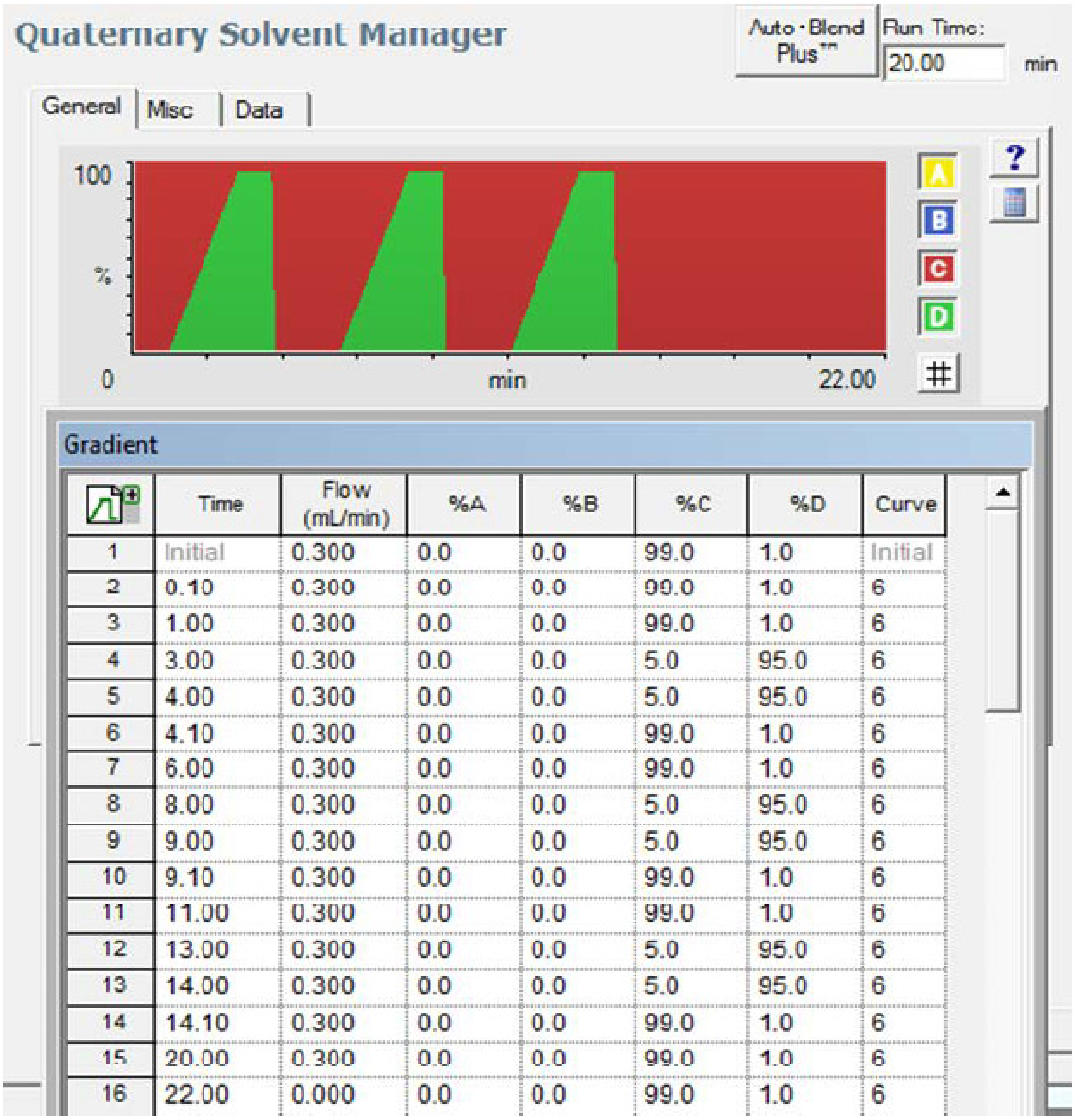

	Time	Flow (mL/min)	%A	%B	%C	%D	Curve
1	Initial	0.300	0.0	0.0	99.0	1.0	Initial
2	0.10	0.300	0.0	0.0	99.0	1.0	6
3	1.00	0.300	0.0	0.0	99.0	1.0	6
4	3.00	0.300	0.0	0.0	5.0	95.0	6
5	4.00	0.300	0.0	0.0	5.0	95.0	6
6	4.10	0.300	0.0	0.0	99.0	1.0	6
7	6.00	0.300	0.0	0.0	99.0	1.0	6
8	8.00	0.300	0.0	0.0	5.0	95.0	6
9	9.00	0.300	0.0	0.0	5.0	95.0	6
10	9.10	0.300	0.0	0.0	99.0	1.0	6
11	11.00	0.300	0.0	0.0	99.0	1.0	6
12	13.00	0.300	0.0	0.0	5.0	95.0	6
13	14.00	0.300	0.0	0.0	5.0	95.0	6
14	14.10	0.300	0.0	0.0	99.0	1.0	6
15	20.00	0.300	0.0	0.0	99.0	1.0	6
16	22.00	0.000	0.0	0.0	99.0	1.0	6

Supplement S5. Optimized MS method for multiple reaction monitoring (MRM) analysis of ACT, DNT and TCT analysis on the Xevo™ TQ-S.

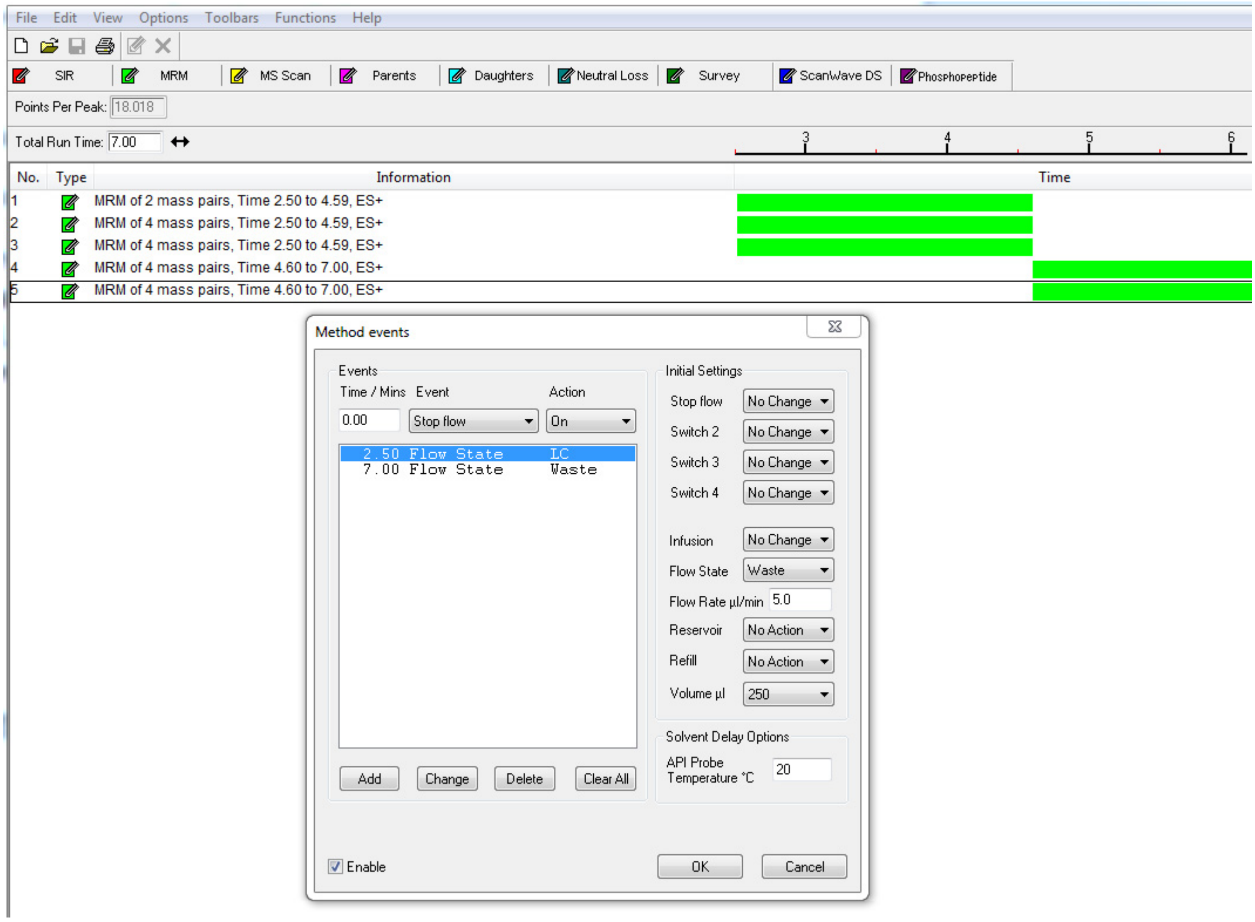

MRM Function	Peptide	Fragment	Transition	Description	Precursor (m/z)	Fragment (m/z)	Cone (V)	Collision Energy (V)
1	TCT	Loss of GlcNAc	1	TCT	922.3888	719.3099	35	31
					461.6985	719.3099	35	11
2	NIENAVGSAR (ACT)	y8	1	Native	515.7674	803.4006	35	19
				Heavy AQUA	520.7716	813.4089	35	19
		y7	2	Native	515.7674	674.3580	35	22
				Heavy AQUA	520.7716	684.3663	35	22
3	ITGDAQANVLR (ACT)	y9	1	Native	579.3173	943.4956	35	22
				Heavy AQUA	584.3214	953.5038	35	22
		y5	2	Native	579.3173	572.3515	35	22
				Heavy AQUA	584.3214	582.3597	35	22
4	ELPALIGASGLR (DNT)	y6	1	Native	598.8535	560.3151	35	21
				Heavy AQUA	603.8576	570.3234	35	21
		y7	2	Native	598.8535	673.3991	35	24
				Heavy AQUA	603.8576	683.4074	35	24
5	NDDLVSIAATYDR (DNT)	y8	1	Native	726.8519	896.4472	35	24
				Heavy AQUA	731.8560	906.4555	35	24
		b3	2	Native	726.8519	345.1041	35	29
				Heavy AQUA	731.8560	345.1041	35	29

Supplement S6. TCT fragment ions from high-energy collision induced dissociation (HCD) on a Q-Exactive HF Orbitrap mass spectrometer.

Precursor (m/z)	Fragment (m/z)	Description	Ion Type	Chemical Formula
922.3893 (+)<br>461.6985 (++)	922.3853	Intact TCT molecular ion $[M+H]^+$	Precursor	$[C_{37}H_{60}O_{20}N_7]^+$
	719.3073	Loss of GlcNAc	Peptide	$[C_{29}H_{47}O_{15}N_6]^+$
	701.2969	Product from loss of GlcNAc + oxygen	Peptide	$[C_{29}H_{47}O_{14}N_6]^+$
	534.2393	Product of glycan cleavage (loss of 1-6-anhydro sugar GlcNAc + MurNAc)	Peptide	$[C_{21}H_{36}O_{11}N_5]^+$
	516.2292	Product of glycan cleavage	Peptide	$[C_{21}H_{34}O_{10}N_5]^+$
	445.1918	Peptide fragment	Peptide	$[C_{18}H_{29}O_9N_4]^+$
	391.1813	Peptide fragment (y-ion)	Peptide	$[C_{15}H_{27}O_8N_4]^+$
	302.1339	Peptide fragment	Peptide	$[C_{12}H_{20}O_6N_3]^+$
	262.1392	Peptide fragment (Ala-DAP) y2 ion	Peptide	$[C_{10}H_{20}O_5N_3]^+$
	204.0862	Intact GlcNAc oxonium ion	Glycan	$[C_8H_{14}O_5N]^+$
	173.0918	HexNAc fragment ion	Glycan	$[C_7H_{11}O_4N]^+$
	138.0547	Oxonium fragment ion	Glycan	$[C_7H_8O_2N]^+$
	126.0549	Oxonium fragment ion	Glycan	$[C_6H_8O_2N]^+$
	239.1021, 329.1336, 630.2602	Consistent with fragment ions (MS/MS) from TCT peptidoglycan homologue in *Neisseria gonorrhoeae* (2)	N/Ap	Not defined

Supplement S7. ACT AQUA peptide dose linearity with linear (solid line) and quadratic (dotted line) fits from 1 to 100 fmol on-column.

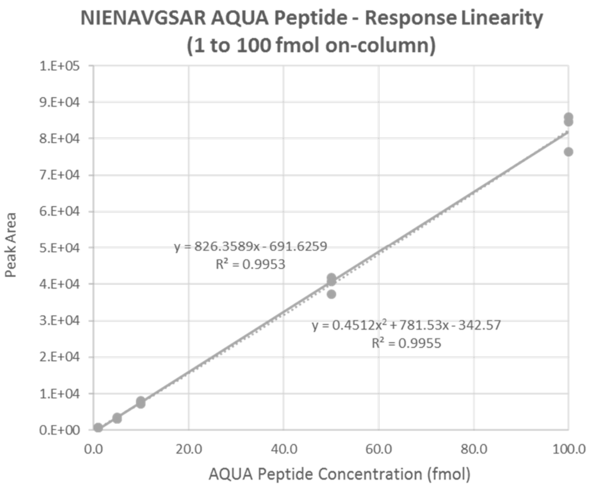

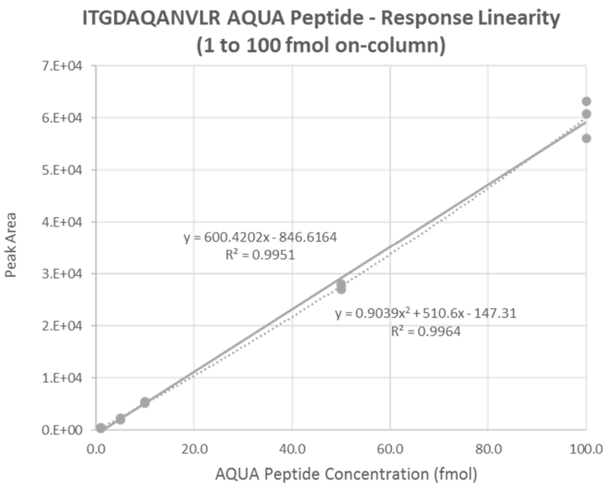

Supplement S8. External TCT calibration curves using the singly and doubly charged fragment ion transitions (922.40 > 719.31 and 461.70 >719.31) for MRM analysis.

a) Linear – 5.31 to 531 pg

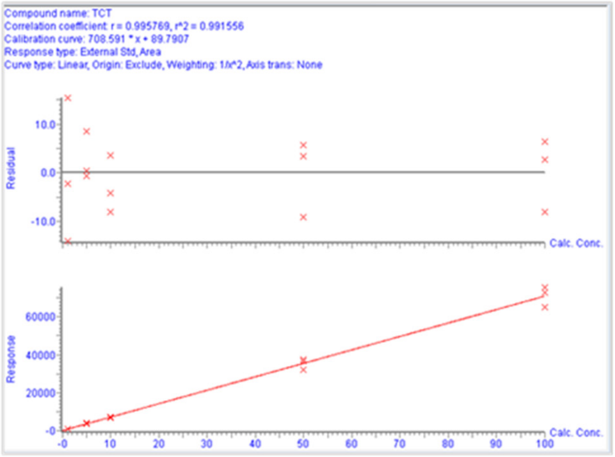

b) Quadratic – 5.31 to 531 pg

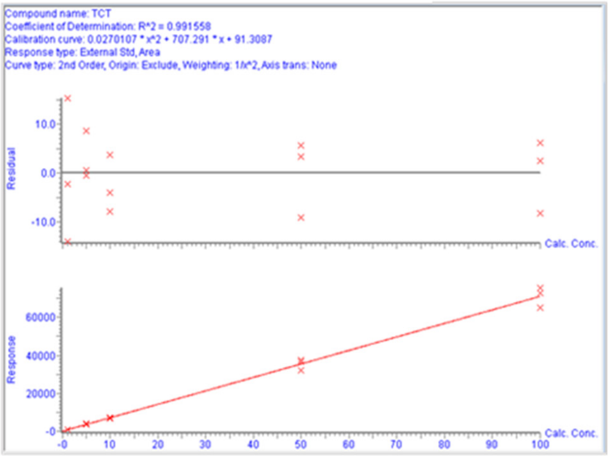

c) Linear – 5.31 to 5310 pg

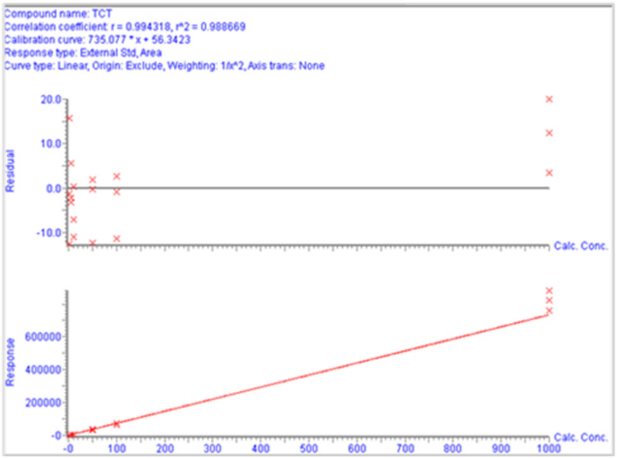

d) Quadratic – 5.31 to 5310 pg

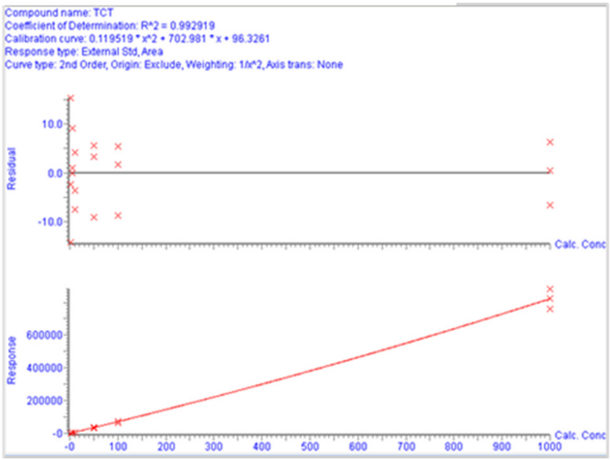

Supplement S9. TCT signal response from a multi-sample external calibration curve analyzed by MRM. Signal response from 922.40 > 719.31 and 461.70 >719.31 transitions denoted by TCT 1+ and TCT 2+, respectively.

TCT (pg)	TCT 1+		TCT 2+		TCT 1+ & TCT 2+		Ratio of TCT 2+ / TCT 1+	
	Average Peak Area (n=3)	% CV	Average Peak Area (n=3)	% CV	Average Peak Area (n=3)	% CV	Average Ratio	% CV
**5.31**	1.91E+02	13.9%	5.98E+02	14.5%	7.89E+02	13.8%	3.14	**10.2%**
**26.6**	9.45E+02	17.9%	2.79E+03	8.3%	3.74E+03	4.7%	3.04	**24.6%**
**53.1**	1.72E+03	4.3%	5.26E+03	8.7%	6.98E+03	6.1%	3.07	**11.1%**
**266**	8.74E+03	3.7%	2.68E+04	11.8%	3.55E+04	8.0%	3.07	**15.1%**
**531**	1.70E+04	1.4%	5.42E+04	9.5%	7.12E+04	7.5%	3.19	**8.6%**
**5314**	2.85E+05	5.8%	5.38E+05	8.6%	8.23E+05	7.4%	1.88	**5.0%**

References

1. Scott KB, Turko IV, Phinney KW. Chapter Eleven - QconCAT: Internal Standard for Protein Quantification. In *Kelman Z, editor. Methods in Enzymology [Internet]*. Academic Press; 2016 [cited 2020 Mar 27]. p. 289–303. (Isotope Labeling of Biomolecules—Applications; vol. 566).
2. Martin SA, Rosenthal RS, Biemann K. Fast atom bombardment mass spectrometry and tandem mass spectrometry of biologically active peptidoglycan monomers from Neisseria gonorrhoeae. *J. Biol. Chem.* **1987**,*262*, 7514–7522.
